# Instrumental Variable Analyses in Pharmacoepidemiology: What Target Trials Do We Emulate?

**DOI:** 10.1007/s40471-017-0120-1

**Published:** 2017-10-17

**Authors:** Sonja A. Swanson

**Affiliations:** Department of Epidemiology, Erasmus MC, P. O. Box 2040, 3000 CA Rotterdam, The Netherlands

**Keywords:** Instrumental variable, Target trial, Pharmacoepidemiology

## Abstract

**Purpose of review:**

When leveraging observational data to estimate treatment effects, it is useful to explicitly specify the “target trial” the investigators aspire to emulate. One concern is whether a proposed analysis plan can address the realities of the differences between the available non-randomized observational study and the target trial. When large or unknown sources of unmeasured confounding are suspected, investigators might consider turning to instrumental variable (IV) methods. Of course, the interpretation and appropriateness of IV analyses need to be considered carefully. The purpose of this review is to summarize recent methodologic advancements in how epidemiologists weigh the validity of an IV analysis and to place these methodologic advancements in the context of the feasible target trial’s protocol components.

**Recent findings:**

There have been increased development and application of tools for sensitivity analyses, falsification strategies, and the identification of previously overlooked problems with IV analyses as applied in pharmacoepidemiology. Many of these recent insights can be seen as articulating restrictions on or tradeoffs between the types of target trials that can be validly emulated when using a classical IV analysis.

**Summary:**

Putting classical IV methods in the context of target trials underscores the importance of recent methodologic developments and, more generally, when and how an IV analysis would be appropriate. We see that some tradeoffs in defining the target trials are unavoidable, that some tradeoffs may be offset or explored via sensitivity analyses, and that this serves as a framework for scientific discourse regarding IV and non-IV results emulating potentially different trials with different tradeoffs.

## Introduction

When leveraging observational data to estimate treatment effects, it is useful to explicitly specify the “target trial” the investigators aspire to emulate [1••]. Indeed, clearer and more valid causal inferences can be achieved by specifying key components of the target trial protocol: eligibility criteria, treatment strategies, assignment procedures, follow-up period, outcome, causal contrast of interest, and analysis plan.

Of course, one of the concerns of investigators emulating target trials is whether their proposed analysis plan can address the realities of the differences between their non-randomized observational study and the target trial. When large or unknown sources of unmeasured confounding are suspected, common analytic approaches may not be successful in even approximately emulating the random assignment procedures of the specified target trial, and investigators might consider turning to instrumental variable (IV) methods as a complementary or alternative analysis plan. Indeed, as confounding by indication and related biases are a major concern in pharmacoepidemiology, the attraction of IV and related methods that do not require measuring confounders is obvious [2].

While a number of resources have discussed the appropriateness and interpretation of IV analyses [2–7, 8•, 9•], the use of IV methods in observational studies is infrequently put in the context of the target trial’s protocol. Here, we take this opportunity to discuss how specifying the target trial’s eligibility criteria, treatment strategies, assignment procedures, follow-up period, outcome, and causal contrast of interest can inform the plans to use or avoid an IV analysis. Throughout, we refer to timely examples of published pharmacoepidemiologic IV applications to illustrate key points and common practices, and we put recent methodologic advancements and insights in the context of feasible target trials’ protocol components. As we will see below, deep consideration of the target trial provides additional context regarding the importance of these methodologic developments and, more generally, when and how an IV analysis would be appropriate. Namely, by specifying the target trial of interest, we can see whether an IV analysis can ever be valid for it, and, if yes, how to perform the IV analysis appropriately to do so (Table 1). We begin with a brief review of core IV concepts.

**Table 1:** Summary of key components of the target trials that, in principle, can be emulated in observational data using methods that adjust for measured confounders compared to classical instrumental variable methods

Target trial feature	Target trials that can be emulated using methods that adjust for measured confounders	Target trials that can be emulated using classical instrumental variable methods
Eligibility criteria	No specific restrictions	When estimating a “local” effect, being a “complier” is an additional eligibility criterion (note: this further criterion would be impossible to evoke in a real trial)
Treatment strategies	No specific restrictions (can emulate trials of point interventions or sustained treatment strategies)	Can only emulate trials of point interventions (including initiating a sustained treatment strategy) Ignoring some of the indicated treatment strategies existing in the data requires stronger assumptions
Assignment procedures	Can only emulate random assignment without blinding	Can only emulate random assignment without blinding
Follow-up period	No specific restrictions	No specific restrictions
Outcome	No specific restrictions (frequently, blind ascertainment cannot be emulated)	No specific restrictions (frequently, blind ascertainment cannot be emulated)
Causal contrast of interest	No specific restrictions (can be used to estimate the effect of initiating or sustaining a sustained treatment strategy, or the effect of a point intervention)	Can be used to estimate the effect of a point intervention (including the effect of initiating a sustained treatment strategy), but generally not to estimate the per-protocol effect of sustaining a treatment strategy

## Core Instrumental Variable Concepts

IV methods allow estimation of treatment effects when a pre-treatment variable, known as an instrument, is available that meets three conditions: (i) it is associated with treatment, (ii) it causes the outcome only through treatment, and (iii) its effect on the outcome is not confounded. Note, the first condition can be checked empirically while the second and third conditions are unverifiable. Usually, more than just an instrument is needed to estimate causal effects, including some form of a homogeneity assumption to estimate an effect in the entire study population or a monotonicity assumption to estimate an effect in a subset; implications related to the latter conditions are described in more detail below.

To illustrate, consider the canonical analysis conducted by McClellan and colleagues [10], which is sometimes described as the first IV analysis in medical research. The authors proposed distance to care as an instrument to study the effect of catheterization following acute myocardial infarction on mortality. As such, they were suggesting that the distance that patients live from medical centers that offered the catheterization treatment would be associated with receiving catheterization, would not have an effect on mortality except though catheterization, and would not share causes with mortality. The second instrumental condition could be violated if distance to care also affected access to other treatments that have effects on mortality other than the treatment of interest; the third instrumental condition could be violated if neighborhoods closer to hospitals have different socioeconomic conditions than those further away (and socioeconomic conditions affected mortality) [11]. The authors—and readers—need to carefully weigh whether these or other violations of the instrumental conditions are plausible. Along with distance to care, commonly proposed instruments in pharmacoepidemiology include provider preference, calendar time, and geographic variation [12].

In describing IV analyses in the context of target trials, we will focus on target trials suitable for estimating an effect of treatment. Some IV-based analyses are performed with different goals in mind, such as estimating the effect of the proposed instrument or testing for a non-null treatment effect. The current focus is motivated by two observations: first, many proposed instruments in pharmacoepidemiology are non-causal, thereby suggesting the proposed instrument itself may not act as a randomizer; and second, the results of pharmacoepidemiologic studies often focus on comparing treatment effect estimates between IV and non-IV analyses, thereby suggesting the analyses are used to emulate comparable target trials.

## Specifications of the Target Trial

### Eligibility Criteria

Publications that present IV analyses in parallel with other analyses appear to employ similar eligibility criteria. One exception is that IV analyses sometimes require additional eligibility criteria to define the proposed instrument. For example, in order to conduct an analysis with the proposed preference-based instrument “treatment provided to the prior patient seen by the same physician,” one additional eligibility criterion—that the proposed instrument is measured, meaning that each patient is not the first eligible patient treated by their physician in the dataset—is required [13]. To understand whether target trials with or without this additional eligibility criterion would result in very different estimates, some investigators perform their non-IV analyses with and without this restriction to see whether effect estimates for these analyses meaningfully change.

However, while the eligibility criteria for an IV and non-IV analysis emulating a target trial may appear overtly similar, there is an enormously important difference between such analyses. In order for the IV analysis to emulate the same exact target trial with the same exact eligibility criteria as a non-IV analysis based on adjusting for measured confounders, investigators must either make heroic (and often biologically implausible) homogeneity assumptions [2, 14] or only compute bounds based on the instrumental conditions alone [14, 15]. Rather, most IV analyses use a different assumption—monotonicity—in order to estimate a “local” effect that only pertains to a subset of the study population [16]. In other words, evoking a monotonicity condition means the target trial’s eligibility criteria are necessarily restricted further; moreover, in defining these further restrictions, we will realize they would generally be impossible to instigate.

So, who belongs to this subpopulation? They are the “compliers,” a subgroup defined with respect to counterfactual treatment levels based on levels of the causal instrument [16]. In a given study, suppose we conducted three IV analyses with three different proposed instruments: one based on calendar time, one based on geographic variation, and one based on provider preference. Because the definition of a “complier” is instrument-dependent [17], the “compliers” in these three analyses would not be the same. Even if all three proposed instruments were indeed instruments, at best, the three IV analyses are estimating effects in three trials with different eligibility criteria—and with different eligibility criteria than a non-IV analysis conducted in the same dataset [18].

The implications are more than simply imposing different eligibility criteria, however. How would we describe the eligibility criteria here? We cannot know who the “compliers” are at baseline. As such, being a “complier” is not a criterion we could evoke in a trial [19, 20]. However, there are some mitigating factors. For some analyses (but not all [21]), we may be able to leverage measured data to characterize the “compliers” to some degree [9•, 16, 22, 23•]. Thus, while we cannot specify the target trial eligibility criteria a priori, it is sometimes possible afterwards to describe which study participants were more likely to have been eligible for the target trial. Augmenting studies with a survey of providers suggest that this characterization might be improved further in the case of preference-based instruments [23•, 24].

Altogether, it is difficult to underscore enough how different this type of “local” effect is from the target trial emulation concept. Mitigating factors aside, we can never pre-specify a well-defined trial that could estimate the same causal effect as is being estimated in these IV analyses, which hinders both interpretability and usefulness of the results of an IV analysis under monotonicity [20]. For readers not convinced that a lack of a well-defined target trial is a limitation, the same reasoning implies such IV analyses cannot directly inform treatment decisions for a well-defined population. This poses a major limitation of this type of IV analysis, as pharmacoepidemiologic studies are often explicitly motivated by questions regarding clinical or public health decision-making.

### Treatment Strategies

Specifying a target trial requires detailed information on the treatment strategies in each arm of the trial. Treatment strategies of interest in pharmacoepidemiology could be point interventions (e.g., “receive a one-time influenza vaccination”) or a sustained treatment strategy (e.g., “take antidepressant medication daily for six months or until contraindications arise”). Unfortunately, classical IV methods are usually inappropriate for studying the effects of adhering to sustained treatment strategies: classical IV methods are restricted to target trials comparing time-fixed or point treatments [2]. As such, the treatment strategies studied with classical IV methods involve a one-time medical procedure or the initiation of a treatment.

Arguably, this restriction to target trials that compare time-fixed treatment strategies severely limits the scope of IV methods. While the general theory of g-estimation of structural nested models supports estimation of effects of sustained treatment strategies if a time-varying instrument is available, such methods are not typically applied and would require detailed knowledge about the treatment’s relationship with the outcome [25]. As many if not most key public health questions involve sustained treatment strategies, the placement of IV analyses in epidemiologists’ toolbox serves at best a limited or complementary role.

Among the possible point treatment strategies that could be compared, there is another refinement to consider. One possible set of treatment strategies could be to compare initiating a specific treatment (or class of treatments) to not initiating that treatment. Another possible set of treatment strategies could be to compare initiating one available treatment to another available treatment. In the first set, all eligible individuals fall into one of the treatment strategies of interest, but in the second set, they do not. Based on a recent review of IV applications [3], perhaps, the majority of applications fall into this latter category. As examples, comparisons across antipsychotic medication classes [26–28], anti-inflammatory medication classes [29, 30], specific antidepressant medications [31], and cancer therapies [32] have all been made with IV analyses; in each of these examples, it is possible that “no treatment” or treatment with other therapies would also be indicated.

This brings us to another point in which IV and non-IV analyses used to emulate the same target trial can diverge. For successful emulation of a trial with either set of treatment strategies, non-IV analyses based on adjusting for measured confounders would be similar in either case: although the particular covariate adjustments could differ, the analyses would be agnostic to whether the comparison involves an exhaustive set versus only a subset of available treatment strategies. The comparison of a non-exhaustive set of treatment strategies (e.g., a comparison of two types of statin medications while ignoring the realistic alternative option of taking neither statin) in a classical IV analysis could result in large and counterintuitive biases due to selecting on a subset of available treatment strategies [33•]. Unless investigators can argue why this bias is implausible or of minimal concern in their particular study, an option would be to augment the IV analysis with specific inverse probability weights using measured covariates [14, 33•, 34]. However, these weights would essentially need to incorporate confounders of treatment: that is, the very set of unmeasured confounders that perhaps motivated conducting an IV analysis may in fact be the reason why an IV analysis comparing active treatments would also be biased [33•]. Another option in some cases would be to conduct an IV analysis comparing more than two treatment strategies in order to conduct an analysis on an exhaustive set of strategies; of course, this would require having a valid instrument for the expanded research question. Tools to investigate the possible magnitude or direction of resulting bias when selecting on treatment have been developed in recent years [33•, 35, 36].

### Assignment Procedures

It is nearly always impossible to emulate target trials with blinded assignment in observational data, as typically both the individuals and their healthcare providers are aware of the treatments prescribed [1••]. This is regardless of whether an IV approach is taken. Thus, the target trial that can be emulated is akin to some pragmatic trials [37].

To emulate random assignment, the typical non-IV approaches (e.g., propensity score analyses; inverse probability weighting) require adjustment for all baseline confounders needed to ensure conditional exchangeability of the groups defined by initiating each treatment strategy. IV approaches replace this exchangeability condition with the instrumental conditions, i.e., they require that the proposed instrument is indeed an instrument. Given that the causal conclusions of any target trial analysis rely on successful emulation of random assignment, it is imperative that study investigators and readers carefully weigh the plausibility of the assumptions required for emulating random assignment and understand the robustness of their conclusions to realistic violations of each assumption.

While epidemiologists have decades of practice thoughtfully discussing and empirically investigating the exchangeability assumption of typical non-IV analyses [38–40], many of the available falsification strategies, sensitivity analyses, and means for evaluating the instrumental conditions have only recently been developed or adopted. One promising practice that is more common in non-IV analyses [41] is to use negative control outcomes that are not expected to be affected by the treatment to see whether an IV analysis of this negative outcome indeed finds the anticipated null result [42]. Another practice that indirectly examines the third instrumental condition is to compare covariate balance of measured covariates across levels of the proposed instrument (under the assumption that unmeasured covariates would be similarly balanced). While such covariate balance assessments are employed even in the earliest epidemiologic IV applications [10], more recently, covariate balance assessments augmented by the strength of the proposed instrument have been proposed [43] and put into practice [13, 42] in order to avoid misleading comparisons to non-IV approaches. Relatedly, investigators can turn to subject matter knowledge and empirical evidence (perhaps published from other data sources or study populations) to identify possibly sources of confounding that violate the third instrumental condition [11]; in fact, having external estimates of the associations between an unmeasured covariate, the proposed instrument, and the outcome along with the proposed instrument’s strength could inform a quantitative bias analysis for the IV estimate [6, 43]. Finally, it is worth noting that, while we cannot ever know that the instrumental conditions hold, we can sometimes find evidence against them based on computing the instrumental inequalities [15, 44, 45]. To date, there are still few applied papers that explicitly demonstrate the instrumental inequalities are satisfied or use other such falsification strategies [3].

### Follow-Up Period

When emulating a target trial with pharmacoepidemiologic data, the follow-up period begins at some unified “time zero” at which eligibility criteria are met, treatment is assigned, and outcome recording begins [1••]. For any observational data analysis, aligning these features of “time zero” can prevent many potential biases [46–48].

Outcome recording then continues for a specified length of follow-up. Some IV applications have follow-up periods as short as a few months (as in a study of antidepressant medication treatments and self-harm [31]) or as long as several years (as in a study of colorectal cancer treatments and survival [32]). The length of the follow-up period has two important implications to IV and non-IV analyses emulating the same trial.

First, with longer follow-up periods comes more opportunity for losses to follow-up to occur. It is an important but oft-overlooked fact that IV methods do not protect against loss to follow-up or related selection biases [34, 49•, 50–52]. Like their non-IV counterparts, the analytic procedure should often be augmented to try to address these potential biases explicitly (e.g., by incorporating inverse probability of censoring weights). It does not appear that such analytic procedures are commonly employed in published IV analyses, although there has been recent methodological literature supporting these practices [49•].

Second, noting the follow-up period helps put in context whether studying initiating (rather than maintaining) a sustained treatment strategy is of public health, clinical, or personal decision-making relevance. With longer follow-up periods, it is more likely that members of the study population diverge from the initiated treatment strategy.

### Outcome

Ideally, a target trial would include blind ascertainment of the outcome, or systematic measurement of the outcome, in order to ensure treatment status itself does not influence recording of the outcome. When using electronic medical records or administrative claims to ascertain outcomes, usually we cannot emulate this kind of target trial because we cannot guarantee that the provider recording the outcomes is unaware of the patient’s treatment status. (Blind ascertainment could be convincingly emulated if, for example, the outcome is death independently ascertained from a mortality registry.) Outcome ascertainment that is not blinded can be problematic for any analysis if the treatment indeed affects the recording of the outcome of interest. For an IV analysis, biases could also arise if the proposed instrument affects the recording of the outcome. For example, if calendar time was proposed as an instrument, it could be problematic if outcome ascertainment changed over time because of new diagnostic tools or procedures.

### Causal Contrast of Interest

Trial protocol specifications also include the causal contrasts of interest. When describing feasible treatment strategies, we noted that classical IV analyses are generally restricted to the study of time-fixed or point interventions. Thus, examples of causal contrasts studied in IV applications include contrasting the initiation of certain medications [26, 29, 32] or contrasting the receipt of one-time medical procedures such as vaccination or surgery [10, 53, 54]. In the latter case, this can be conceived as the per-protocol effect in a trial assigning these point interventions. In the former case when the treatment strategies of interest are sustained treatment strategies, the IV analysis will only be appropriate for studying causal contrasts of initiating the treatment strategies, while other methods could be used to also study causal contrasts of initiating and adhering to sustained treatment strategies.

## Conclusions

Hernán and Robins [1••] argued that even when emulating an ideal trial is not feasible, the target trial approach is nonetheless useful as it “allows us to systematically articulate the tradeoffs that we are willing to accept.” Putting classical IV methods in this context indeed illuminates tradeoffs that are unavoidable, such as only considering time-fixed treatment strategies [2]. It also puts in context the tradeoffs we may offset in future study designs or explore via sensitivity analyses [21, 23•, 42, 44]. Finally, it provides a framework for scientific discourse regarding IV and non-IV results emulating potentially different trials with potentially different tradeoffs [20, 55].

In sum, restricting oneself to conducting IV analyses in the appropriate class of target trials already rules out a number of biases and concerns. From there, it is up to the investigators to diligently weigh remaining tradeoffs in formulating a suitable target trial and—if they decide to pursue an IV approach for emulating that trial—to then conduct an appropriate analysis, understand the robustness of the conclusions from the analysis, and triangulate the results as feasible.
